# Multiple Squamous Cell Carcinoma in a Patient Using Skin Bleaching Products in Togo

**DOI:** 10.1155/2023/8002896

**Published:** 2023-01-11

**Authors:** Abas Mouhari-Toure, Panawé Kassang, Winga Foma, SefakoAbla Akakpo, Julienne Noude Teclessou, Kelly Tcheumagam, Essobiziou Amana, Kwame Doh, Gloria Nouhoumon, Tchin Darre, Koussake Kombate, Palokinam Pitché, Bayaki Saka

**Affiliations:** ^1^Service de Dermatolgieet IST, CHU Kara, Université de Kara, Kara, Togo; ^2^Service de Dermatolgieet IST, CHU SylvanusOlympio, Université de Lomé, Lomé, Togo; ^3^Service ORL, CHU SylvanusOlympio, Université de Lomé, Lomé, Togo; ^4^Service de Dermatolgieet IST, CHU CampusUniversité de Lomé, Lomé, Togo; ^5^Laboratoired'anatomieetcytotologiepathologique, CHU SylvanusOlympio, Université de Lomé, Lomé, Togo

## Abstract

**Background:**

The cosmetic use of skin bleaching products is common among women in sub-Saharan Africa despite numerous reported cutaneous and systemic complications. We report the first case of squamous cell carcinoma in a woman using skin bleaching products in Togo. *Case Report*. A 65-year-old woman with a 30-year history of skin bleaching products use consulted in dermatology for a tumor of the neck that had been evolving for 2 years. There was no personal or family history of cancer. The patient was obese (BMI = 38.3 kg/m^2^) and had high blood pressure. Clinical examination noted multiple ulcerative and cauliflower tumors of the neck. The presence of stretch marks, skin atrophy, and ochronosis was noted in the examination of the rest of skin. There were no lymph nodes. HIV serology was negative. Histology of a tumor biopsy concluded to an invasive skin squamous cell carcinoma. The cervical, thoracic, abdominal, and pelvic TDM revealed pulmonary metastases. The patient underwent complete surgical removal of the right latero-cervical tumor. The left latero-cervical tumors were not removed because they infiltrated the large vessels. Chemotherapy before surgery was prescribed but not honored for financial reasons. The patient died 2 months after her first consultation in respiratory distress.

**Conclusion:**

Squamous cell carcinoma is one of the complications of skin bleaching in sub-Saharan Africa. It is necessary to intensify awareness campaigns on the complications of this practice, in order to reduce their incidence, in our context where this practice is very frequent.

## 1. Introduction

Skin bleaching can be defined as all practices leading to cosmetic depigmentation of the skin. It is a practice by a person, on his or her own initiative, who seeks to reduce or eliminate the physiological pigmentation of the skin [1]. This cosmetic use of lightening products is common in sub-Saharan black populations with the prevalence ranging from 25% to 77.3% according regions [2–4]. In a recent study, the main factors associated with these skin bleaching products use were as follows: age, income level, initial skin color, existence of own income, housing status, diabetes, high blood pressure, and overweight or obesity [4]. Skin bleaching has cutaneous and systemic consequences of varying severity depending on the products and the duration of their use [3, 5]. Squamous cell carcinoma associated with the use of skin bleaching products has long been reported in sub-Saharan Africa [6–10], but no case in Togo, in our knowledge. Herein, we report a case of squamous cell carcinoma in a woman using skin bleaching products in Togo.

## 2. Case Report

A 65-year-old woman, a phototype VI of Fitzpatrick's classification, consulted in dermatology for multiple ulcerative and cauliflower tumors of the neck. The time from onset of tumors to consultation was two years. The initial lesion was a tumor in the left cervical region which became ulcerated, followed two months later by the occurrence of two other tumors on the left and right side of the neck. The patient had received treatment with antibiotics, antiseptics, and traditional herbal poultices without success. Topical hydroquinone and highly potent corticosteroids such as clobetasol propionate were the main products used on the whole body and the face for a duration of 30 years. There is no personal or family history of cancer. The patient was obese (BMI = 38.3 kg/m^2^) and was known to be hypertensive.

**Figure 1:**
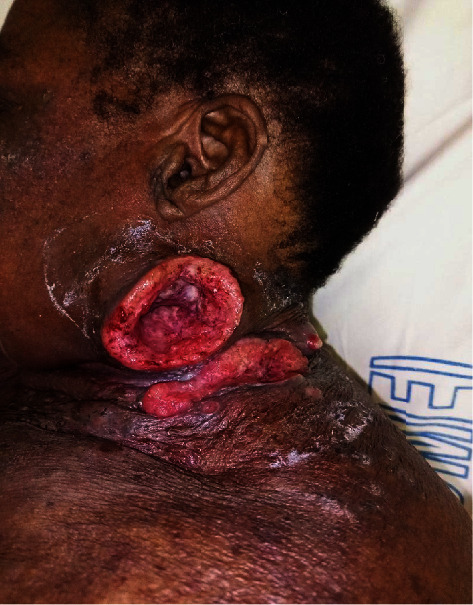
Left latero-cervical ulcerative tumors.

**Figure 2:**
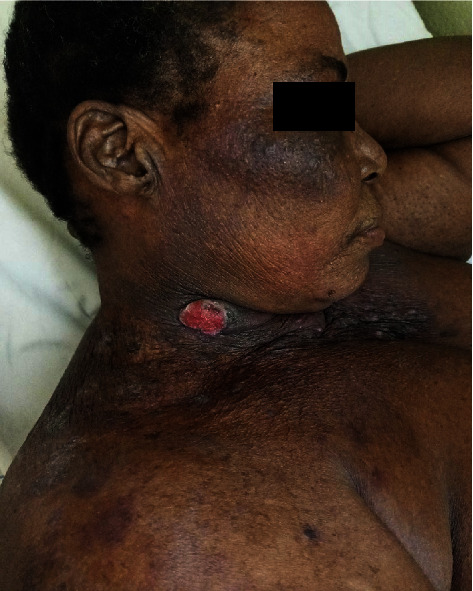
Right latero-cervical ulcerative and cauliflower tumor.

On examination, there were three painful, bleeding on contact, left latero-cervical ulcerative tumors with an infiltrated base measuring 6 × 5 cm, 3 × 3 cm, and 8 × 2 cm, respectively (Figure 1), and an ulcerative tumor measuring 4 × 3 cm on the right latero-cervical side (Figure 2). Angiomatous nodular lesions were also present in the vicinity of these tumors and on the anterior aspect of the neck. The rest of the skin examination showed exogenous ochronosis of the neck and face, and diffuse skin atrophy with local ecchymotic patches and stretch marks on the whole body. There were no associated palpable lymph nodes. The rest of the clinical examination was normal.

**Figure 3:**
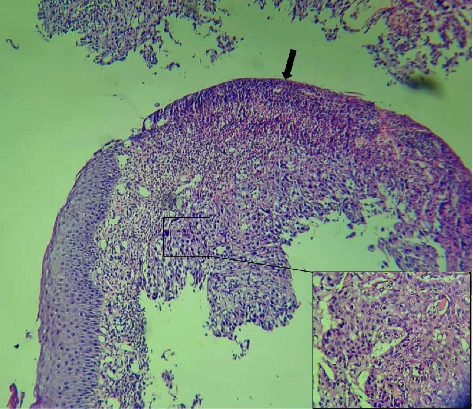
Histological appearance of a moderately differentiated squamous cell carcinoma. *Note*: Under an ulceration (arrow), a squamous cell proliferation best seen on the cartridge (HE, x20; Cartridge x40).

Blood tests for viral hepatitis and human immunodeficiency virus (HIV) were negative. The pathological examination of tumor biopsy concluded to an invasive skin squamous cell carcinoma (Figure 3). The cervical, thoracic, abdominal, and pelvic TDM revealed pulmonary metastases. The patient underwent a complete surgical removal of the right latero-cervical tumor. The left latero-cervical tumors were not removed because they infiltrated the large vessels. Chemotherapy before surgery was prescribed but not honored for financial reasons. The patient died 2 months after her first consultation in respiratory distress.

## 3. Discussion

We report the first case of skin squamous cell carcinoma in a woman using skin bleaching products in Togo. The particularity of our observation lies in the multiple and large ulcerated character of the tumors. Such multiple and large ulcerated lesions were probably due to the long duration of use of very highly potent skin bleaching products. Except this multiple and large ulcerated character, all the epidemiological and anatoclinical characteristics of our patient were comparable to those of the cases reported in the literature [6, 8, 9].

Epidemiologically, the case reported by Addo [9] was a 58-year-old Ghanaian woman, and the ages of the 8 cases reported by Ly et al. [8] ranged from 37 to 68 years. The patient in our case was 65 years old, in contrast to the young age of 30 years reported in the observation of Faye et al. [6]. The age of onset of lesions is related to the early onset of skin bleaching products, since the delay between the beginning of skin bleaching and the onset of lesions varied from 10 to 30 years. Clinically, the delay in diagnosis, the localization in photo-exposed areas especially in the neck, and the clinical presentation in the form of ulcerating lesions have been reported by other authors [6, 8]. The delay in diagnosis explains the presence of pulmonary metastases and the evolution towards death in 3 months in our patient. In the series by Ly et al. [8], one patient had bone metastases and two deaths, one of which was due to metastases. This delay in consultation also explains the fact that the histology showed an infiltrating squamous cell carcinoma in our study, as in the series of Faye et al. [6] and in most of the observations of Ly et al. [8] (7 patients out of 8) versus only one case of carcinoma in situ.

Several arguments make us suspect the role of skin bleaching products in our patient: the absence of family or personal history of skin cancer, the high phototype, and the absence of any known risk factor for squamous cell carcinoma such as HPV infections, paraneoplastic dermatoses, and genodermatoses. Several mechanisms can explain how squamous cell carcinoma develops in our patient: the oncotic actions of hydroquinone [11, 12] and the UV light because in most patients, the sun exposed areas are the only site affected. Also, dermocorticoids used in the long term have systemic and local side effects such as skin atrophy and local immunosuppression which may play a role in skin carcinogenesis [3, 5]. The combination of these three factors therefore seems to play a major role in the development of cancers in skin bleaching [13]. In view of these data, fundamental studies should be conducted to establish a clear pathophysiological basis for the occurrence of skin cancers in depigmentation areas. In parallel, African countries should find other solutions, in addition to awareness raising, to ban the import, and production and distribution of skin bleaching products such as Cameroon [14].

## 4. Conclusion

Skin bleaching is a common practice in our countries and skin disorders secondary to these practices are increasingly reported. In addition to awareness raising, new preventive measures must be put in place not only by the health authorities but also by the competent authorities in charge of the legislation regulating cosmetics in Togo.

## Abbreviations

UV: Ultraviolet.
